# Biomarkers in Interstitial Cystitis/Bladder Pain Syndrome with and without Hunner Lesion: A Review and Future Perspectives

**DOI:** 10.3390/diagnostics11122238

**Published:** 2021-11-30

**Authors:** Yoshiyuki Akiyama

**Affiliations:** Department of Urology, Graduate School of Medicine, The University of Tokyo, Tokyo 1138655, Japan; yakiyamauro-tky@umin.ac.jp; Tel.: +81-35-800-8753; Fax: +81-35-800-6435

**Keywords:** interstitial cystitis, bladder pain syndrome, IC/BPS, PBS, biomarker

## Abstract

Interstitial cystitis/bladder pain syndrome (IC/BPS) is a debilitating urinary bladder condition that presents with a wide variety of clinical phenotypes. It is commonly characterized by persistent pelvic pain and lower urinary tract symptoms, such as urinary frequency and urgency. Current clinicopathological and genomic evidence has indicated that IC/BPS with Hunner lesions is a clinically relevant distinct subtype with proven bladder pathology of subepithelial chronic inflammatory changes that are characterized by enhanced local immune responses and epithelial denudation. However, other forms of IC/BPS lacking Hunner lesions are a symptom syndrome complex of non-inflammatory conditions with little evidence of bladder etiology, characterized by aberrant neural activity in neurotransmission systems which leads to central nervous sensitization with potential involvement of urothelial malfunction, or clinical presentation of somatic and/or psychological symptoms beyond the bladder. Given such distinct potential pathophysiology between IC/BPS subtypes, disease biomarkers of IC/BPS should be provided separately for subtypes with and without Hunner lesions. Tailored approaches that target characteristic immunological inflammatory processes and epithelial denudation for IC/BPS with Hunner lesions, or the sensitized/altered nervous system, urothelial malfunction, association with other functional somatic syndromes, and psychosocial problems for IC/BPS without Hunner lesions, are essential to identify optimal and reliable disease-specific IC/BPS biomarkers.

## 1. Introduction

Interstitial cystitis/bladder pain syndrome (IC/BPS) is a symptom syndrome complex characterized by persistent pain that is perceived to be related to the urinary bladder in conjunction with urinary frequency and/or urgency, and presents with a wide variety of clinical phenotypes [1,2,3,4]. Although the complete picture of IC/BPS remains unclear, current evidence has revealed that there is a clinically relevant subtype of IC/BPS: a Hunner lesion subtype [4,5]. This subtype is diagnosed by a cystoscopic marker, known as the Hunner lesion, which is a reddish mucosal lesion that is accompanied by abnormal capillary structures [1]. Recent clinicopathological and genomic evidence has indicated that IC/BPS with Hunner lesions is a robust immunological inflammatory disease of unknown origin in the bladder, characterized by frequent clonal expansion of infiltrating B cells, pancystitis, and epithelial denudation. Meanwhile, IC/BPS without Hunner lesions is a symptom syndrome complex of non-inflammatory conditions that frequently presents with non-bladder symptoms, such as somatic and/or psychological symptoms [6,7,8,9,10]. It is potentially associated with neuroendocrine dysfunction, psychosocial problems, and genetic predisposition, which ultimately lead to the hypersensitivity of neurotransmission systems. Currently, IC/BPS with and without Hunner lesions are widely recognized as entirely distinct disease entities with different pathophysiology and clinical manifestations [5].

A well-recognized and clinically relevant disease marker of IC/BPS is the Hunner lesion. However, recognition of the Hunner lesion can be challenging because of its varied appearance [11]. Moreover, diagnoses of Hunner lesions are made by urologists who perform cystoscopy in a subjective manner. Thus, there is a crucial need to identify less invasive and more precise and objective disease markers for IC/BPS; however, reliable and reproducible disease biomarkers remain elusive. This challenge may be attributed to past biomarkers studies for IC/BPS exploring multiple clinical conditions of IC/BPS (i.e., pathogenetically different entities) as a single cohort. In this article, we discuss the potential biomarkers of IC/BPS separately for each subtype of IC/BPS and suggest future strategies to identify optimal IC/BPS disease biomarkers.

## 2. Disease Concept and Classification of IC/BPS

Recently, a clinical rationale disease concept of IC/BPS was developed by European, North American, and East Asian groups in terms of its clinical characteristics, epidemiology, and bladder histology [1,12,13]. Consensus was reached whereby IC/BPS with Hunner lesions was proven to be a clinically relevant subtype that should be regarded as a distinct disease entity. However, other forms of IC/BPS without Hunner lesions have not yet been clearly defined owing to their obscure pathophysiology, histology, and varied clinical manifestations. Thus, classifying IC/BPS into the following two subtypes is reasonable at present: IC/BPS with Hunner lesions, which is also known as BPS type 3 based on the International Society for the Study of BPS (ESSIC) criteria; and IC/BPS without Hunner lesions, which includes ESSIC BPS types 1 and 2 [1,2,3]. These two subtypes with or without Hunner lesions are distinguished simply by the cystoscopic presence or absence of Hunner lesions; however, they cannot be discerned by clinical phenotyping because of the overlap in clinical characteristics [14]. Clinically, compared with IC/BPS without Hunner lesions, IC/BPS with Hunner lesions is characterized by an older age of onset, more severe bladder-centric symptoms, diminished bladder capacity, fewer comorbid non-bladder syndromes, and more favorable outcomes upon endoscopic treatment [15,16]. IC/BPS without Hunner lesions is frequently accompanied by non-bladder symptoms, which include other systemic pain problems, personal psychosocial health problems, and affect dysregulation [17]. Recent studies have suggested a possible link between IC/BPS without Hunner lesions and widely known somatoform disorders or functional somatic syndromes (FSSs), such as irritable bowel syndrome, fibromyalgia, chronic fatigue syndrome, and migraines [18]. A subgroup of patients with IC/BPS without Hunner lesions may present with an FSS, which manifests as somatic symptoms related to the bladder. Moreover, bladder histology is distinct between the two IC/BPS subtypes; IC/BPS with Hunner lesions has proven bladder histology, showing epithelial denudation and chronic inflammatory changes such as lymphoplasmacytic and mast cell infiltration, stromal fibrosis, and edema [9,19]. Notably, these histological changes can be observed in the entire bladder (i.e., pancystitis) and are not confined to the Hunner lesion [6]. By contrast, IC/BPS without Hunner lesions shows few histological changes [6,19]. Such histological evidence indicates that IC/BPS with Hunner lesions is a robust chronic inflammatory disease of the bladder, whereas IC/BPS without Hunner lesions is a non-inflammatory condition with no obvious bladder etiology. In addition, a recent ribonucleic acid sequencing (RNA-seq) study revealed that whole transcriptomic profiles differ between these two subtypes. IC/BPS with Hunner lesions features enhanced biological processes that involve immune responses and infection, whereas, the gene expression profiles of the bladder of IC/BPS patients without Hunner lesions were similar to those of normal bladders, and no specific biological pathways were detected in this subtype [8].

Glomerulations (mucosal bleeding after bladder overdistension) has been considered as another characteristic disease marker of IC/BPS. However, recent studies have cast doubts on its significance in the diagnosis and etiology of IC/BPS [20]. To investigate the biological background of glomerulation formation, we performed genomic and histological analyses of bladder biopsy samples from patients with IC/BPS without Hunner lesions, with or without glomerulations. We revealed that the presence of glomerulations did not alter gene expression patterns in the bladder mucosa and was not associated with the degree of subepithelial inflammation or neovascularization [8], which suggests that glomerulations is not a specific disease marker of IC/BPS. However, we currently cannot conclusively discount its significance in IC/BPS, and further validation using independent resources is necessary to resolve this controversy.

Taken together, the current IC/BPS syndrome umbrella should be considered to consist of two subtypes: IC/BPS with Hunner lesions, which is an inflammatory disease of the bladder; and IC/BPS without Hunner lesions, a non-inflammatory condition of the bladder that may involve pathology beyond the bladder.

## 3. Biomarkers of IC/BPS

Table 1 lists the potential biomarkers of IC/BPS with and without Hunner lesions.

### 3.1. Immunological Inflammatory Process in the Bladder

Characteristic inflammatory features of IC/BPS with Hunner lesions include the accumulation of plasma cells and the frequent expansion of light-chain-restricted B cells in the bladder [5,6]. Previously, we conducted an RNA-seq analysis of mucosal biopsies of IC/BPS and healthy control bladders. Results indicated that genes involved in biological pathways related to immune responses and infectious diseases were significantly up-regulated in IC/BPS with Hunner lesions. In addition, we found a tumor necrosis factor family B-cell-activating factor (BAFF) as a potential biomarker of IC/BPS with Hunner lesions [8]. These findings collectively suggest that an immunological inflammatory process that is potentially associated with a B-cell abnormality may underlie the pathophysiology of IC/BPS with Hunner lesions. Candidates for potential biomarkers of IC/BPS, especially for the Hunner lesion subtype, may be identified in genes/proteins that are involved in this immunological inflammatory process.

#### 3.1.1. Chemokines

There have been reports of increased expression of several chemokines in the bladder and urine of patients with IC/BPS with Hunner lesions [21,22,23]. Tyagi et al. found that urinary levels of C-X-C motif chemokine ligand (CXCL) 1 and CXCL10 were significantly increased in IC/BPS patients with Hunner lesions compared with those of patients without Hunner lesions and asymptomatic control patients [24]. Furuta et al. reported that urinary levels of CXCL8 and CXCL10 were significantly increased in IC/BPS patients with Hunner lesions than in patients without Hunner lesions and in patients with overactive bladder syndrome (OAB) [25]. In addition, Niimi et al. revealed that urinary levels of CXCL10 were significantly increased in patients with Hunner lesions, and these levels correlated with symptom severity. They demonstrated that CXCL10 urine concentration is a potential disease biomarker with modest sensitivity (46.1%) and high specificity (93.7%) [26]. These findings suggest that CXCL10 could be a potential disease biomarker of IC/BPS with Hunner lesions.

Another candidate chemokine as a biomarker of IC/BPS is C-C motif chemokine ligand 2 (CCL2), also known as monocyte chemotactic protein-1 (MCP-1). Previous studies have reported increased levels of urinary CCL2 in patients with IC/BPS [25,27,28]. In 16 women with IC/BPS, Peters et al. reported a positive correlation between urinary levels of CCL2 and daily urinary frequency and treatment responsive changes. Urinary levels of CCL2 decreased significantly from baseline values at 24 weeks after sacral neuromodulation therapy [28].

#### 3.1.2. Proinflammatory Cytokines

Elevated levels of various cytokines, such as interleukin (IL)-1, IL-6, and IL-8, have been reported in patients with IC/BPS, which reflects its inflammatory nature [29,30,31,32,33]. IL-6 is produced by various immune cells, which include T helper type 2 (Th2) cells, mast cells, and macrophages, and plays a pivotal role in inflammatory reactions, such as the induction of Th2 immune responses, antibody production, and mast cell recruitment. Elevated urinary IL-6 levels are related to the degree of nocturnal urinary frequency in patients with IC/BPS [31]. Combined with methylhistamine, urinary levels of IL-6 have a sensitivity of 70%, a specificity of 72.4%, a positive predictive value of 77.8%, and a negative predictive value of 63.6% in the diagnosis of IC/BPS [32]. Matrix metalloproteinases (MMPs) play an important role during inflammatory processes, such as tissue remodeling, cytokine release, angiogenesis, and recruitment of inflammatory cells [34,35]. A study that investigated biomarkers of urological chronic pelvic pain syndrome (UCPPS) using urine samples of 259 patients with UCPPS and 125 healthy controls indicated that urinary levels of MMP-9 were significantly increased in male UCPPS patients compared with those of controls, and were related to pain and urinary symptom severity [36]. Recently, IL-17 has emerged as a novel inflammatory cytokine that is potentially associated with various autoimmune diseases [37]. As for IC/BPS with Hunner lesions, Logadottir et al. reported the overexpression of IL-17 in both messenger RNA (mRNA) and protein in the bladder of IC/BPS patients with Hunner lesions [30]. Moreover, our RNA-seq analysis indicated that IL-17-related biological pathways were more up-regulated in the bladders of IC/BPS patients with Hunner lesions than in the bladders of those without Hunner lesions and normal controls [8]. In psoriasis, anti-IL-17 therapies have been developed and applied clinically [38]. Further studies are required to validate whether IL-17 has the potential to become a novel biomarker and therapeutic target for IC/BPS with Hunner lesions.

#### 3.1.3. Toll-like Receptor

A previous study reported that elevated inflammatory responses by Toll-like receptor (TLR)2 and TLR4 stimulation in peripheral blood mononuclear cells (PBMCs) were observed in 24 female IC/BPS patients, and the degree of elevation was associated with changes in pain score and urinary symptoms [39]. Another study demonstrated that compared with healthy control patients’ bladders, TLR7 was more expressed in the bladders of IC/BPS patients with Hunner lesions [40]. Both studies also verified the rationales of the study results using murine models; altered TLR4 activation was associated with bladder nociception and voiding dysfunction in autoimmune cystitis female mice [41], and activation of TLR7 induced cystitis that was accompanied by sensory hyperactivity of the bladder in C57BL/6N female mice [40]. Furthermore, a study by the Multidisciplinary Approach to the Study of Chronic Pelvic Pain (MAPP) research network demonstrated that higher inflammatory responses to TLR4 stimulation in PBMCs are significantly associated with a greater likelihood of endorsing widespread (bladder-beyond) pain, a higher number of comorbid conditions, and lower pain thresholds in patients with IC/BPS [42]. The authors offered TLR4 inflammatory responses in PBMCs as a marker of the presence and degree of widespread pain in IC/BPS.

#### 3.1.4. Angiogenic Factors

Increased levels of vascular endothelial growth factor (VEGF), a proinflammatory growth factor that induces neovascularization, have been reported in patients with IC/BPS with Hunner lesions [8,36,43]. We demonstrated the overexpression of the VEGF protein and increased microvessel density in the bladders of IC/BPS patients with Hunner lesions compared with those in normal bladders [8]. Studies have reported increased nitric oxide production in both the bladder and urine of IC/BPS patients with Hunner lesions [30,44,45]. Furthermore, the overexpression of hypoxia-inducible factor 1α (HIF1α) in Hunner lesions of the IC/BPS bladders has been reported [46]. Increased levels of platelet-derived endothelial cell growth factor (PD-ECGF), which facilitates angiogenesis, have also been reported in the urine of IC/BPS patients [47,48]. This evidence collectively reflects the increased angiogenesis in the bladders of IC/BPS patients, which likely results from chronic inflammatory processes. To determine the potential for these angiogenic factors as disease biomarkers of IC/BPS, specificity must be validated by comparison with non-IC chronic cystitis.

### 3.2. Neurogenic Inflammation

#### Mast Cell Infiltration

Mast cell infiltration is considered a characteristic histological feature of IC/BPS [49,50,51,52,53,54,55,56]. However, emerging evidence has cast doubt on the significance of mast cell infiltration in IC/BPS [57,58,59,60]. We evaluated mast cell densities in IC/BPS with and without Hunner lesions by comparing equally inflamed bladders using an anti-tryptase antibody specific to human mast cells. Results indicated that IC/BPS with Hunner lesions and non-IC chronic cystitis showed similar degrees of subepithelial lymphoplasmacytic infiltration, and healthy and IC/BPS patients without Hunner lesions bladders had a comparable degree of mast cell infiltration [60]. Other studies have also suggested that increased mast cell density may not be a specific histological feature of IC/BPS [57,58,59].

However, the functional role of infiltrated mast cells in IC/BPS cannot be discounted. Enhanced release of neuropeptides from peripheral nerves results in persistent afferent nerve sensitization and local inflammatory changes, which are referred to as neurogenic inflammation [61], and these processes are mediated by mast cells. Neurotransmitters released by peripheral neurons, which include calcitonin gene-related peptide, tachykinins, and substance P, induce mast cell degranulation and the release of proinflammatory mediators from mast cells, such as histamine, serotonin, tryptase, tumor necrosis factor (TNF)α, and nerve growth factor (NGF). Subsequently, these inflammatory mediators respond to afferent neurons in a positive feedback loop, which perpetuates and exacerbates the further release of neuropeptides and mast cell activation (i.e., the nerve–mast cell interaction axis) [62,63,64,65]. Persistent afferent nerve stimulation results in altered neural plasticity and central nervous sensitization in the dorsal root ganglia and upper spinal cord, which contributes to IC/BPS symptom persistence, especially in patients without Hunner lesions [66]. It is well established that mast cells are implicated in other disorders characterized by afferent hypersensitivity and neurogenic inflammation that frequently overlap those of IC/BPS without Hunner lesions [67,68,69,70]. In this context, previous reports of elevated NGF levels in patients with IC/BPS are unsurprising [22,27,71,72]. Liu et al. reported that both urinary and serum levels of NGF were significantly elevated in patients with IC/BPS compared with those of healthy subjects; however, these did not correlate with clinical characteristics [71]. Moreover, Homma et al. reported that mRNA levels of NGF were more elevated in the bladders of IC/BPS patients without Hunner lesions than in the bladders of non-IC controls, although the levels of NGF did not correlate with symptom parameters [22]. These results indicate the relatively low power of NGF as a disease biomarker of IC/BPS. Overexpression of TNFα in the bladder, and elevated serum and urinary levels of TNFα in IC/BPS patients, have been reported previously [72,73]. A randomized, double-blind, placebo-controlled study of anti-TNFα monoclonal antibody (certolizumab pegol) treatment demonstrated significant symptom improvement in patients with IC/BPS compared with that in patients who received placebo [74].

### 3.3. Urothelial Deficiency

A urothelial defect is generally considered one of the cardinal etiologies of IC/BPS. Previously, potential biomarkers of IC/BPS have been suggested via this hypothesis. Disruption of the urothelium permits subnoxious urinary irritants to permeate into the subepithelial layer, which results in the elicitation of inflammatory reactions or afferent nerve stimulation. Notably, urothelial alterations, which are undetectable by optical microscopy, can also impair the barrier function and contribute to increased permeability. This enables urinary stimuli to gain access to the subepithelial tissue and to persistently stimulate afferent neurons, which leads to central nervous amplification with subtle histological changes [75].

#### 3.3.1. Glycosaminoglycans

The glycosaminoglycans (GAG) layer plays a crucial role in the protective barrier function of the urothelium. Disruption of the GAG layer has been observed in the urine of patients with IC/BPS [76]. Urinary uronate and sulfated GAG levels have been shown to be increased in patients with IC, with 80% and 88% sensitivity, respectively, and 92.3% and 69.2% specificity, respectively, for detecting IC symptom severity [77].

#### 3.3.2. Growth Factors for Epithelium

Aberrant urothelial proliferation, which may be due to the altered production of antiproliferative or growth factors, has been reported in patients with IC/BPS [78,79]. Previous studies have indicated that levels of epidermal growth factor (EGF), insulin-like growth factor-1, and insulin-like growth factor-binding protein-3 are elevated in the urine of patients with IC/BPS [80,81,82,83]. In particular, antiproliferative factor (APF), which plays a cardinal role in inhibiting bladder epithelial cell proliferation, has been extensively studied and is increased in the urine of patients with IC/BPS. In fact, the level of APF activity is considered to be the most reliable urine biomarker of IC/BPS [80,84,85,86,87]. Keay et al. compared urinary APF activity and levels of heparin-binding (HB)-EGF and EGF among 219 IC patients, 113 asymptomatic healthy control patients, and 211 patients with other non-IC urogenital diseases, such as acute bacterial cystitis, chronic prostatitis, OAB, bladder cancer, and prostate cancer. Results indicated that APF activity is significantly higher in IC patients than in all other control groups, with 94% sensitivity and 95% specificity [84]. However, HB-EGF, which plays a crucial role in bladder epithelial metabolism by counteracting APF, was down-regulated in the bladders of IC/BPS patients [80,82,83]. Interestingly, previous studies reported that the decreased urinary HB-EGF levels and increased APF activity were reversed by bladder hydrodistension to normal levels in patients with IC/BPS, which may explain the mechanism of action underlying bladder hydrodistension treatment for IC/BPS [88,89]. As for the comparison of these markers between IC/BPS with and without Hunner lesions, Zhang et al. reported that urinary APF activity and HB-EGF concentrations were significantly more altered in IC/BPS patients both with and without Hunner lesions than in those with bladder cancer and bacterial cystitis and healthy controls, although the two subtypes of IC/BPS were comparable. However, levels of urinary EGF were significantly higher in IC/BPS patients with Hunner lesions than in those without Hunner lesions. Given the current understanding of the pathophysiology of IC/BPS with and without Hunner lesions mentioned above, the aberrant proliferative function of urothelial cells may be a common feature of the two subtypes, albeit via different underlying mechanisms.

APF activity may be associated with the down-regulation of epithelial tight junction proteins, such as E-cadherin and zonula occludens-1 (ZO-1). One study suggested that E-cadherin and ZO-1 were lower in IC/BPS patients without Hunner lesions than in those with stress urinary incontinence or OAB [90]. Keay et al. reported that blocking the antiproliferative activity of asialylated APF using inactive APF derivatives reversed the decrease in the level of mRNA and protein expression of tight junction proteins, such as ZO-1, occludin, and claudins, as well as the increase in the paracellular permeability of IC/BPS epithelial cells, back to normal levels [91].

Taken together, a combined panel of urinary APF activity, HB-EGF, and EGF would achieve higher diagnostic accuracy for IC/BPS. Further studies assessing their levels in other lower urinary tract dysfunctional diseases, such as OAB or chronic prostatitis, are warranted to confirm the utility and specificity of these candidates as potential disease biomarkers of IC/BPS.

### 3.4. Nociceptive Reflux Pathways

Increased gene expression of transient receptor potential (TRP) channels, which mediate nociceptive sensation, has been reported in IC/BPS patients. Homma et al. demonstrated that mRNA levels of TRP ankyrin 1, TRP melastatin 2 and 8, and TRP vanilloid subtype 1 were significantly higher in the bladders of IC/BPS patients with Hunner lesions than in those of non-IC controls, whereas mRNA levels of TRP vanilloid subtype 2 and NGF were higher in IC/BPS patients without Hunner lesions [22]. These findings suggest that there is increased activation of the nociceptive reflux pathways in IC/BPS patients. Comparisons with other sensory bladder disorders, such as OAB or chemical cystitis, may provide support for the expression levels of these sensory-mediating channels as potential biomarkers of IC/BPS.

### 3.5. Somatic Symptoms

Growing evidence suggests a potential connection between IC/BPS and FSSs. One study reported that the presence of somatic symptoms significantly increases the risk of the subsequent occurrence of IC/BPS without Hunner lesions [92]. Furthermore, a study that performed magnetic resonance imaging of the brain of patients with UCPPS and fibromyalgia and of pain-free controls demonstrated similar abnormal brain activity in patients with UCPPS and fibromyalgia [93]. These findings suggest that a subgroup of IC/BPS patients may share pathogenetic neurophysiological abnormalities that involve the central nervous system with patients with FSSs [94]. These aberrant neuroimmune and endocrine processes that are responsible for central nervous sensitization and systemic hypersensitivity (i.e., the potential underlying pathophysiology of IC/BPS and FSSs) may offer clues toward identifying disease biomarkers of IC/BPS [95].

## 4. Summary and Future Perspectives

In this article, we first emphasized the crucial differences between IC/BPS with and without Hunner lesions. IC/BPS with Hunner lesions is a robust inflammatory disease of the urinary bladder that is potentially associated with enhanced immune responses and infection, whereas IC/BPS without Hunner lesions is a symptom syndrome complex of non-inflammatory conditions with little evidence of bladder etiology and is potentially associated with aberrant neuroimmune and/or endocrine processes. Thus, the current IC/BPS umbrella does not represent the spectrum of a single disorder; rather, it comprises distinct disorders. In this context, it is reasonable and ideal that investigations of potential biomarkers of IC/BPS are conducted separately according to the subtypes of with or without Hunner lesions. Some previous biomarker studies for IC/BPS have been conducted without a clear differentiation between IC/BPS subtypes, which may have eluded true results. Based on subtype specificity, the potential biomarkers of IC/BPS are BAFF, CXCL10, HIF1α, IL-17, and TLR7 for IC/BPS with Hunner lesions, and NGF and PD-ECGF for IC/BPS without Hunner lesions (Table 1). To develop clinically applicable and reliable biomarkers, these candidates should be validated using independent sources and eligible counterparts. For example, bacillus Calmette–Guérin-induced cystitis and follicular cystitis manifest clinicopathological features that are similar to those of IC/BPS with Hunner lesions, including cystoscopic reddish mucosal lesions, histological epithelial denudation, and dense lymphoplasmacytic infiltration, which are frequently accompanied by lymph follicles and pelvic pain. To confirm the specificity of the abovementioned candidates as biomarkers of IC/BPS with Hunner lesions, comparative analyses of the levels of these candidates with non-IC/BPS chronic inflammatory cystitis are needed. Likewise, comparisons of candidate marker levels between IC/BPS without Hunner lesions and OAB and FSS are warranted. Biomarkers with higher sensitivity and specificity for both subtypes of IC/BPS, the current confusion in the definition, diagnosis, and classification of IC/BPS would be relieved. In terms of clinical application, urine biomarkers that could specifically diagnose IC/BPS and the subtypes, and also reflect the treatment responses should be ideal. A tailored approach that targets characteristic immunological inflammatory processes and epithelial denudation for IC/BPS with Hunner lesions, or the sensitized/altered nervous system, urothelial malfunction, associations with other FSSs, and psychosocial problems for IC/BPS without Hunner lesions, may lead to better outcomes in future investigations of potential biomarkers of IC/BPS.

## Figures and Tables

**Table 1:** Potential biomarkers of IC/BPS.

Candidate	Subtype †	Specific Notes	Reference
APF	Unspecified	Increased urinary APF activity and related changes in intensity levels to bladder distension [88,89]	[84,85,86,87,88,89]
BAFF	HL	Overexpression of BAFF protein in the HL bladder	[8]
CCL2	HL and NHL	Elevated urinary levels of CCL2 in HL and/or NHL compared with those in OAB [25,27], and association with treatment response of neuromodulation [28].	[25,27,28]
CXCL10	HL	Urinary levels of CXCL10 discriminated HL from NHL and correlated with symptom severity [26]	[24,25,26]
EGF	Unspecified	Significantly higher urinary levels of EGF in IC/BPS than in non-IC conditions	[80,81,82,83]
HB-EGF	Unspecified	Lowered urinary levels of HB-EGF in IC/BPS and increased by therapeutic hydrodistension [88,89]	[80,82,83,88,89]
HIF1α	HL	Up-regulation of HIF1α in mRNA and protein levels in the HL bladder	[8,46]
IL-6	Unspecified	Elevated urinary IL-6 levels in IC/BPS and correlation with symptom severity [31]	[31,32,72,73]
IL-17	HL	Overexpression of IL-17A in the HL bladder	[8,30]
MMP-9	Unspecified	Elevated urinary levels of MMP-9 in UCPPS and association with clinical symptoms	[36]
NGF	NHL	Increased levels of NGF in serum [71,72], urine [27,71], and bladder [22] of NHL	[22,27,71,72]
NO	HL	Up-regulation of mRNA and protein levels of NO products in the HL bladder	[30,44,45]
PD-ECGF	NHL	Increased urinary levels of PD-ECGF and high association with bladder glomerulations [48]	[47,48]
TLR4	Unspecified	Increased response to TLR4 stimulation in PBMC of IC/BPS patients and significant association with symptom changes and spread	[39,42]
TLR7	HL	Overexpression of mRNA and protein of TLR7 in the HL bladder	[40]
TNFα	Unspecified	Increased levels of TNFα in the serum [72] and urine [73] of IC/BPS patients	[72,73]
VEGF	HL	Overexpression of VEGF in the HL bladder [8] and significant association between urinary levels of VEGF and clinical symptoms in UCPPS [36]	[8,36,43]

† HL: Hunner lesion subtype (IC/BPS with Hunner lesions), NHL: non-Hunner lesion subtype (IC/BPS without Hunner lesions).

## Abbreviations

APF	antiproliferative factor
BAFF	tumor necrosis factor family B-cell-activating factor
CCL2	chemokine (C-C motif) ligand 2
CXCL10	chemokine (C-X-C motif) ligand 10
EGF	epidermal growth factor
ESSIC	International Society for the Study of BPS
FSS	functional somatic syndrome
GAG	glycosaminoglycan
HB-EGF	heparin-binding growth factor-like growth factor
HIF1α	hypoxia-inducible factor 1α
IC/BPS	interstitial cystitis/bladder pain syndrome
MMP	matrix metalloproteinase
NGF	matrix metalloproteinase
PD-ECGF	platelet-derived endothelial cell growth factor
TLR	Toll-like receptor
UCPPS	urological chronic pelvic pain syndrome
VEGF	vascular endothelial growth factor
